# Tinea genitalis profunda caused by *Microsporum canis*: A case report and literature review

**DOI:** 10.1016/j.mmcr.2025.100738

**Published:** 2025-09-23

**Authors:** Samet Öner, Ceylan Avcı

**Affiliations:** Dokuz Eylül University Hospital, Department of Dermatology, 35330, İzmir, Türkiye

**Keywords:** Tinea profunda, Kerion, Majocchi granuloma, *Microsporum canis*, Genital dermatophytosis, Inflammatory tinea

## Abstract

Deep dermatophytosis in the genital region is rare and often misdiagnosed due to its inflammatory presentation mimicking bacterial or other dermatoses. We report the case of a 23-year-old woman presenting with painful, exudative plaques in the pubogenital area. Initial empirical antibiotic therapy failed. Fungal culture revealed Microsporum canis. Treatment with systemic terbinafine followed by itraconazole led to clinical improvement. The lesion healed with scarring alopecia and post-inflammatory hyperpigmentation. Given the overlapping features with both kerion and Majocchi granuloma, this case was classified as tinea profunda. This case highlights the importance of considering deep dermatophytosis in the differential diagnosis of genital inflammatory and suppurative lesions, especially when initial treatments fail.

## 1 Introduction

Tinea profunda refers to a deep dermatophyte infection that extends beyond the epidermis into the dermis and subcutaneous tissue. It encompasses a spectrum of clinical entities including kerion and Majocchi granuloma [1,2]. Kerion typically affects the scalp and is characterized by boggy, purulent plaques, while Majocchi granuloma manifests as perifollicular nodules or plaques resulting from follicular invasion. However, the distinction between these two conditions remains unclear, especially in atypical localizations such as the genital region [4]. This case report describes a rare presentation of tinea profunda caused by *Microsporum canis* in a young immunocompetent female, emphasizing the diagnostic and therapeutic challenges.

## 2 Case presentation

A 23-year-old immunocompetent woman presented with a one-month history of painful, itchy, and erythematous plaques that started on the medial aspect of her right thigh and spread to the pubic and perineal regions. The patient reported no recent history of sexual intercourse or mechanical trauma such as shaving or epilation in the pubogenital region. The patient applied topical corticosteroids for several days, but noted progressive worsening of the lesions and therefore discontinued the treatment.

**Fig. 1:**
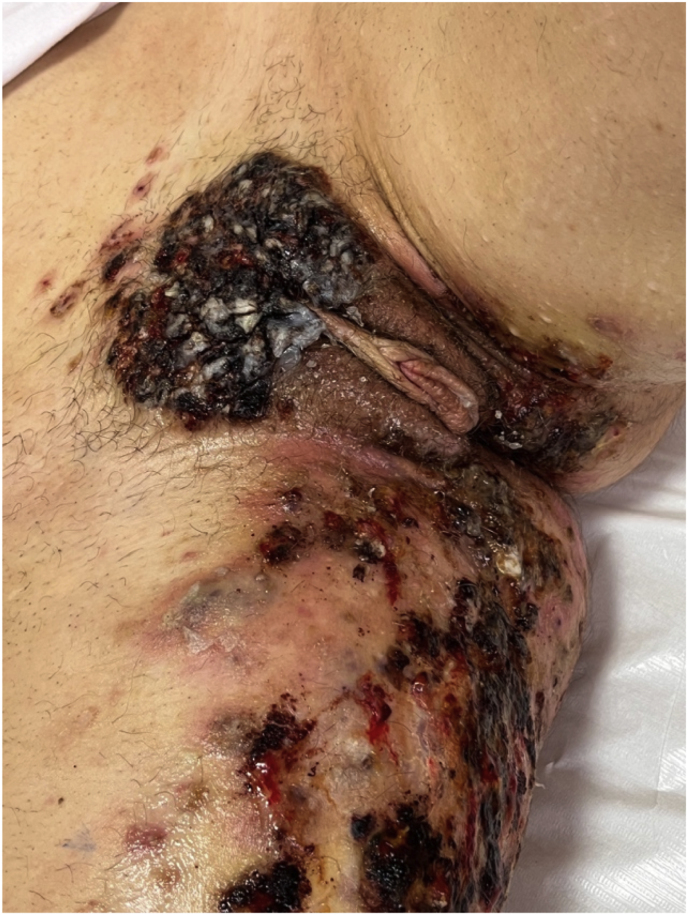
Clinical presentation before treatment: erythematous, exudative plaques and follicular pustules on the mons pubis, labia majora, and right thigh.

On examination, there were extensive erythematous, exudative plaques with hemorrhagic crusting and perifollicular papules and pustules involving the mons pubis, labia majora, and right thigh, consistent with a severe inflammatory dermatosis (Fig. 1). Laboratory findings showed leukocytosis (15.7 x 10^3^/μL), elevated CRP (58 mg/L), and high ESR (48 mm/h). Serological screening for sexually transmitted infections, including syphilis and HIV, were negative.

Non-contrast lower abdominal CT revealed diffuse subcutaneous edema and soft tissue stranding extending from the labium majus to the adductor muscles, without abscess formation. Superficial fluid collections measuring up to 6–7 mm were noted in the medial thigh region. These findings suggested an intense inflammatory process limited to the soft tissue, without evidence of organized abscess or deep bacterial infection.

Superficial tissue ultrasonography demonstrated inguinal lymph nodes, the largest measuring 8×39 mm on the right side, with preserved hilus and benign vascular pattern.

Empirical antibiotic treatment with intravenous clindamycin and ciprofloxacin was initiated but resulted in minimal clinical improvement. Fungal cultures of aspirated material on Sabouraud dextrose agar grew *Microsporum canis*. Upon further questioning, the patient disclosed that she owned a dog, which was fully vaccinated and had no visible skin lesions.

**Fig. 2:**
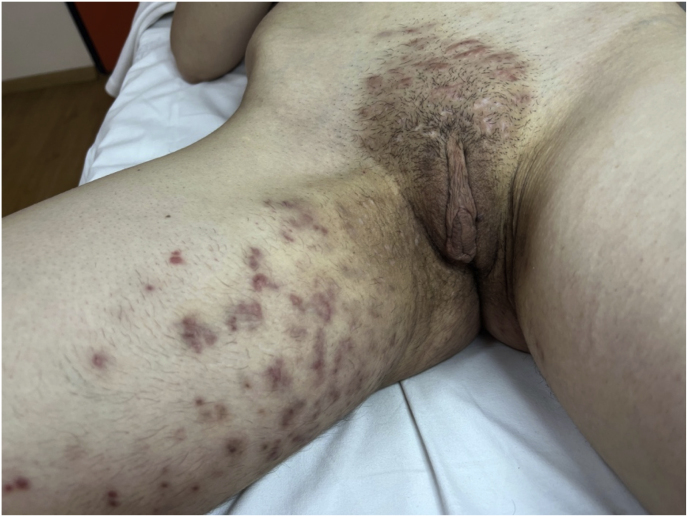
Post-treatment status showing post-inflammatory hyperpigmentation and localized scarring alopecia.

Oral terbinafine (250 mg/day) and topical antifungals were started. This systemic therapy was continued for a duration of 8 weeks. Due to a partial response, systemic therapy was switched to oral itraconazole (200 mg/day). One month after oral itraconazole therapy, the lesions showed marked regression with residual post-inflammatory hyperpigmentation and localized scarring alopecia (Fig. 2).

## 3 Discussion

Tinea profunda encompasses deep dermatophyte infections that penetrate into the dermis and hair follicles, resulting in inflammatory lesions. Two principal clinical variants are kerion and Majocchi granuloma [[1], [2], [3]]. Kerion is usually localized to the scalp and is considered a delayed-type hypersensitivity reaction to zoophilic dermatophytes [2]. Majocchi granuloma, by contrast, typically affects the extremities and is often associated with local trauma or prior topical corticosteroid use, presenting as granulomatous perifollicular inflammation [1,3].

However, recent studies have emphasized the substantial overlap between these conditions. Arenas et al. observed that kerion may present with histopathological features ranging from suppurative folliculitis to granulomatous inflammation, similar to Majocchi granuloma [4]. Likewise, Tirado-Sánchez et al. proposed that these may represent different manifestations along the same pathogenic spectrum [5].

Our patient presented with classic signs of a severe inflammatory dermatophyte infection, including boggy plaques, crusts, and alopecia. Although the clinical appearance resembled kerion, the localization to the pubogenital area and history of topical corticosteroid use also raised the possibility of Majocchi granuloma. Given the overlapping clinical and histopathological features and the absence of definitive histological evaluation in this case, a broader diagnostic label of *tinea profunda* was considered more appropriate.

Radiological imaging supported the diagnosis of an inflammatory fungal process. Non-contrast CT of the pelvis revealed diffuse subcutaneous edema and increased soft tissue density in the medial right thigh and pubic region without signs of an organized abscess, consistent with an intense inflammatory response. Inguinal lymphadenopathy with preserved architecture was also observed, likely reactive in nature. Moreover, superficial ultrasonography demonstrated up to 7 mm fluid accumulations in the dermis and preserved vascularized lymph nodes in the inguinal area. These imaging findings were in line with clinical suspicion of deep dermatophytic folliculitis consistent with tinea profunda, while ruling out bacterial abscesses [6,7].

Additionally, *Microsporum canis*, a zoophilic dermatophyte typically associated with scalp infections, is increasingly recognized as a causative agent of inflammatory tinea in the genital region [6,7]. Gomez-Moyano et al. recently reported three similar cases of “kerion of the pubis” caused by *M. canis* [6], while Ginter-Hanselmayer et al. noted the diagnostic challenges posed by tinea in this region [7]. Ratajczak-Stefańska et al. also described an unusual case of Majocchi’s granuloma due to M. canis in an immunocompetent patient [8]. Several similar cases of genital tinea profunda caused by M. canis have been reported previously and their clinical characteristics, sources of infection, and treatment outcomes are summarized in Table 1. Our case contributes to this emerging evidence and underscores the need for clinicians to consider fungal infections in the differential diagnosis of genital dermatoses.

**Table 1:** Reported cases of tinea genitalis profunda caused by Microsporum canis: Clinical features, infection sources, and treatment outcomes.

Age	Sex	Clinical Findings	Diagnosis	Causative Agent	Source of Infection	Treatment (drug, dose, duration)	Outcome	Reference
23	Female	Erythema, pustules, bald areas and black dots on the pubis	Kerion	Microsporum canis	Not specified	Griseofulvin 500 mg twice daily for 8 weeks	Complete remission and mycological cure	Gomez-Moyano et al., 2025 [6]
14	Female	Edema, erythema, pustules, partial loss of pubic hair	Kerion	Microsporum canis	Not specified	Griseofulvin 25 mg/kg/day for 12 weeks	Patient responded well to treatment	Gomez-Moyano et al., 2025 [6]
36	Male	Swelling and exudation on the pubic area and erythematous desquamative plaques	Kerion	Microsporum canis	Not specified	Griseofulvin 500 mg twice daily for 10 weeks	Complete remission	Gomez-Moyano et al., 2025 [6]
14	Female	Putrid, disseminated, follicularly bound coarse papules on livid erythema	Tinea profunda	Microsporum canis	Anamnestic investigations did not reveal any indications of a possible pathogenesis	Itraconazole (5-week course)	Complete resolution with residual scarring	Ginter-Hanselmayer et al., 2016 [7]
25	Male	Perifollicular papules, pustules, inflammatory nodules on hypogastric area	Majocchi's granuloma	Microsporum canis	Exposure to infected organic waste while working as a courier in an animal shelter	Terbinafine 250 mg/day (8 weeks), cotrimoxazole 480 mg/day (10 days)	Almost complete clinical resolution	Ratajczak-Stefańska et al., 2010 [8]
23	Female	Ertyhematous, exudative plaques with hemorrhagic crusting, perifollicular papules and pustules on the mons pubis, labia majora, and right thigh	Tinea profunda	Microsporum canis	Not specified	Terbinafine 250 mg/day (8 weeks), itraconazole 200mg/day (4 weeks)	Complete resolution with residual scarring	Current case

## 4 Conclusion

This case illustrates a rare and severe form of tinea profunda in the pubogenital region due to *Microsporum canis*, manifesting with inflammatory plaques and partial treatment resistance. It highlights the diagnostic complexity due to overlapping features of kerion and Majocchi granuloma and underscores the importance of fungal cultures in atypical or refractory dermatoses. Early mycological investigation and appropriate antifungal therapy are essential to prevent misdiagnosis and long-term sequelae.

## CRediT authorship contribution statement

**Samet Öner:** Conceptualization, Data curation, Investigation, Visualization, Writing – original draft. **Ceylan Avcı:** Methodology, Supervision, Validation, Writing – review & editing, Project administration.

## Ethical Form

This study received no funding and there are no potential conflicts of interest to declare. Written informed consent was obtained from the patient for publication of this case report and accompanying images.

## Conflict of interest

There are none.
